# Anthocyanin-rich Seoritae extract ameliorates renal lipotoxicity via activation of AMP-activated protein kinase in diabetic mice

**DOI:** 10.1186/s12967-015-0563-4

**Published:** 2015-06-27

**Authors:** Eun Sil Koh, Ji Hee Lim, Min Young Kim, Sungjin Chung, Seok Joon Shin, Bum Soon Choi, Hye Won Kim, Seong Yeon Hwang, Sae Woong Kim, Cheol Whee Park, Yoon Sik Chang

**Affiliations:** Division of Nephrology, The Catholic University of Korea Yeouido St. Mary’s Hospital, 10, 63-ro, Yeongdeungpo-gu, Seoul, 150-713 Republic of Korea; Department of Internal Medicine, College of Medicine, The Catholic University of Korea, 222 Banpo-daero, Seoul, 137-701 Republic of Korea; Division of Nephrology, The Catholic University of Korea Incheon St. Mary’s Hospital, 56, Dongsu-ro, Bupyeong-gu, Incheon, 403-720 Republic of Korea; Division of Nephrology, The Catholic University of Korea Seoul St. Mary’s Hospital, 222 Banpo-daero, Seoul, 137-701 Republic of Korea; Department of Rehabilitation Medicine, Bucheon Saint Mary’s Hospital, Sosa-dong, Wonmi-gu, Bucheon-si, Geoynggi-do 420-717 Republic of Korea; Korea Bio Medical Science Institute, 652, Nonhyeon-ro, Gangnam-gu, Seoul, 135-829 Republic of Korea; Department of Urology, College of Medicine, The Catholic University of Korea, 222 Banpo-daero, Seoul, 137-701 Republic of Korea

**Keywords:** Anthocyanin, AMPK, Diabetic nephropathy

## Abstract

**Background:**

Anthocyanins are major constituents of food colours and have been reported to possess anti-diabetic activities for potential medicinal use. The precise role of anthocyanins in diabetic nephropathy is poorly understood. We investigated whether anthocyanin-rich Seoritae extract (SE) can potentially prevent oxidative stress and lipotoxicity, which are the main causes of renal damage in diabetic nephropathy, via activation of AMP-activated protein kinase (AMPK) and the consequent effects on its target molecules.

**Methods:**

Four groups of male C57BLKS/J *db*/*m* and *db*/*db* mice were used. Diabetic and non-diabetic mice were orally administered 10 mg/kg body weight SE daily for 12 weeks, starting at 8 weeks of age.

**Results:**

*db*/*db* mice treated with anthocyanins showed decreased albuminuria. Anthocyanins ameliorated intra-renal lipid concentrations in *db*/*db* mice with improvement of glomerular matrix expansion and inflammation, which was related to increased phosphorylation of AMPK and activation of peroxisome proliferator-activated receptor (PPAR) α and PPARγ, and inhibited the activity of acetyl-CoA carboxylase and sterol regulatory element-binding protein 1. Anthocyanins reversed diabetes-induced increases in renal apoptosis and oxidative stress. In cultured human glomerular endothelial cells, anthocyanins prevented high glucose-induced oxidative stress and apoptosis through activation of AMPK in the same manner.

**Conclusions:**

The results revealed that anthocyanins ameliorated diabetic nephropathy in *db*/*db* mice via phosphorylation of AMPK, the major energy-sensing enzyme, and the consequent effects on its target molecules, which appeared to prevent lipotoxicity-related apoptosis and oxidative stress in the kidney.

**Electronic supplementary material:**

The online version of this article (doi:10.1186/s12967-015-0563-4) contains supplementary material, which is available to authorized users.

## Background

Diabetic nephropathy is the leading cause of end-stage renal disease and is a growing global health problem. Despite developments in pharmacological strategies to modulate diabetes, diabetic nephropathy remains a major microvascular complication in many patients with diabetes. However, there is still insufficient understanding about the full mechanisms involved in progressive diabetic renal disease. One of the causal factors for the progression of diabetic kidney injury is lipotoxicity. Increasing evidence suggests that renal ectopic lipid accumulation and dysregulated lipid metabolism in the kidney are involved, in part, in the pathophysiology of diabetic nephropathy [1, 2].

Anthocyanins, flavonoids within the polyphenol class, are most abundant in various coloured fruits, vegetables, red wine, and grains, and possess a basic skeleton of 2-phenylbenzopyrylium or flavylium glycoside [3, 4]. Anthocyanins are the most oxidized flavonoids with a fully unsaturated C ring and a hydroxyl at position 3, and are potential therapeutic agents as antioxidants [5]. Their mechanisms of action have been investigated in numerous experimental models in vivo and in vitro. Interest has focused on their potential antioxidant activity, which is proposed as a key mechanism for the prevention of many chronic diseases including metabolic disorders and cancer [6–9]. Anthocyanins have potential antidiabetic activity to protect against pancreatic cell damage and improve insulin sensitivity, and have a potent therapeutic effect on diabetic complications such as diabetic retinopathy [10, 11]. However, it has barely been established whether these natural compounds have beneficial effects on the pathogenesis of diabetic nephropathy.

Adenosine monophosphate-activated protein kinase (AMPK) is a crucial metabolic energy sensor involved in a wide range of biological activities that normalise lipid, glucose, and energy imbalances [12]. AMPK is activated by low energy status (AMP/ATP ratio) and is dysregulated in patients with metabolic syndromes such as diabetes and obesity. It has been reported that AMPK is abundantly expressed in the kidney and its activity is reduced in the diabetic kidney [13]. Experimental evidence suggests that AMPK activation attenuates lipotoxicity, reactive oxygen species generation, inflammation, and endothelial dysfunction in type 2 diabetes [13–15].

The collective results favour the view that anthocyanins have beneficial effects on lipid metabolism dependent on activation of the AMPK pathway in metabolic disorders including type 2 diabetes, obesity, and non-alcoholic fatty liver disease [4, 16]. Anthocyanins ameliorate hyperglycaemia and insulin resistance via activation of AMPK, with inactivation of acetyl-CoA carboxylase (ACC) in hepatocytes, white adipose tissue, and skeletal muscle in diabetic animal models [17].

We hypothesised that anthocyanin-rich Seoritae extract (SE; *Glycine max L.*) may ameliorate renal oxidative stress and lipotoxicity, which are the principal causes of renal damage in diabetic nephropathy, via activation of AMPK and the consequent effects on its target molecules. Glomerular endothelial dysfunction is commonly the initiating insult that predisposes tissue to injury in diabetes [18]. We evaluated the effects of anthocyanins on high glucose-induced oxidative stress and apoptosis related to AMPK activation and its downstream molecules in cultured human glomerular endothelial cells (HGECs).

## Methods

### Preparation of SE and analysis of anthocyanins in SE

The SE used was produced by the following method. Seoritae samples (1,500 g) were extracted with 12,000 mL of 30% ethanol for 3 h at 90–100°C. The solution was filtered twice through 50 and 1-µm filters and concentrated in a vacuum evaporator (60°C) to 70 brix. The residual solvent was removed in a drying machine for 18 h at 60°C in a vacuum. The resulting powder was stored in a plastic bag until use. The anthocyanin content in SE was analysed by HPLC using a Dionex Ultimate 3000 series dual low-pressure ternary gradient pump (Dionex Softron GmbH, Germering, Germany) and an Ultimate 3000 series photodiode array detector for anthocyanin analysis. The crude anthocyanin extract was analysed by its HPLC chromatogram. Three principal anthocyanin peaks were detected in the chromatogram by diode array detection at 530 nm. The major anthocyanins were identified as delphinidin-3-*O*-glucoside (25.2%), cyanidin-3-*O*-glucoside (68.3%), and petunidin-3-*O*-glucoside (6.5%) by comparison with the HPLC retention times of standard compounds, as previously described [19].

### Experimental methods

Male 6-week-old C57BLKS/J *db*/*m* and *db*/*db* mice were purchased from Jackson Laboratories (Bar Harbor, ME, USA). The mice were fed a regular chow diet, provided with water ad libitum, and allowed to acclimatise for 1 week before experiments. The mice were divided into four groups. Control *db*/*m* mice (n = 6) and control *db*/*db* mice (n = 6) received drinking water only, while anthocyanin *db/m* mice (n = 8) and anthocyanin *db*/*db* mice (n = 8) received 10 mg/kg body weight anthocyanin-rich SE daily for 12 weeks. For measurement of 24-h urinary albumin, the mice were placed in individual mouse metabolic cages (Nalgene, Rochester, NY, USA) every 4 weeks. At week 20, the mice were anaesthetised by intraperitoneal injection of a mixture of Rompun (10 mg/kg; Bayer Korea, Ansan, Gyeonggi-Do, Korea) and Zoletil (30 mg/kg; Virbac, Carros, France). Blood was collected from the left ventricle and centrifuged, and the resulting plasma was stored at −70°C for analyses. The kidneys were rapidly dissected and stored in 10% buffered formalin for immunohistochemical analyses. HbA_1c_ was measured from red cell lysates by HPLC (Bio-Rad, Richmond, CA, USA). Triglyceride and total cholesterol concentrations were determined using an automatic analyser (Model 917; Hitachi, Tokyo, Japan) and commercial kits (Wako, Osaka, Japan). NEFA levels were measured with a JCA-BM1250 automatic analyser (JEOL, Tokyo, Japan).

### Ethics statement

All animal experiments were in accordance with the Laboratory Animals Welfare Act, Guide for the Care and Use of Laboratory Animals, and Guidelines and Policies for Rodent Experiments provided by the Institutional Animal Care and Use Committee at the School of Medicine, The Catholic University of Korea (Approval No. YEO20131601FA). All procedures complied with the *Guide for the Care and Use of Laboratory Animals* (National Institutes of Health Publication No. 85–23, revised 1996).

### Assessment of albuminuria, renal oxidative stress, and intra-renal lipid content

Twenty-four-hour urine collection for measurement of albuminuria was performed using metabolic cages (Nalgene) at week 20, and urinary albumin concentrations were obtained by immunoassay (Bayer, Elkhart, IN, USA). To evaluate oxidative stress, we measured 24-h urinary 8-epi-prostaglandin F_2α_ (OxisResearch, Foster City, CA, USA). Intra-renal lipids were extracted using the method of Bligh and Dyer with slight modifications [20].

### Light microscopy study

Kidney samples were fixed in 10% buffered formalin and embedded in paraffin. Histology was assessed after periodic acid-Schiff staining. The mesangial matrix and glomerular tuft were quantified for each glomerular cross-section, as previously reported [21]. More than 30 glomeruli, cut through the vascular pole, were counted per kidney, and the average of the measured areas was used for analysis.

### Immunohistochemistry for TGF-β1, type IV collagen, and TUNEL assay

For immunohistochemistry, 4-μm-thick sections were deparaffinised, hydrated in ethanol, treated with an antigen-unmasking solution containing 10 mmol/L sodium citrate buffer (pH 6.0), and washed with PBS. The sections were incubated with 3% H_2_O_2_ in methanol to block endogenous peroxidase activity, and then with 10% normal goat serum in PBS to block non-specific binding. The sections were incubated overnight with anti-TGF-β1 (1:100; R&D Systems, Minneapolis, MN, USA) and anti-COL IV (1:200; Biodesign International, Saco, ME, USA) antibodies in a humidified chamber at 4°C. The bound antibodies were localised with a peroxidase-conjugated secondary antibody using a Vector Impress Kit (Vector Laboratories, Burlingame, CA, USA) and 3,3-diaminobenzidine substrate solution. Finally, the sections were dehydrated in ethanol, cleared in xylene, mounted without counterstaining, and examined in a blinded manner using light microscopy (Olympus BX-50; Olympus Optical, Tokyo, Japan).

For quantification of proportional areas of staining, ~20 views (×400 magnification) were randomly located in the renal cortex and corticomedullary junction of each section and images were taken. The images were analysed to determine the density X-positive area/glomerular total area using a computer image analysis program (Scion Image Beta 4.0.2; Frederick, MD, USA).

Detection of apoptotic cells in formalin-fixed, paraffin-embedded tissue was performed by in situ TUNEL using an ApopTag In Situ Apoptosis Detection Kit (Chemicon-Millipore, Billerica, MA, USA). The TUNEL reaction was assessed in a whole glomerular biopsy under ×400 magnification.

### Western blot analysis

Total protein from renal cortical tissues was extracted with Pro-Prep Protein Extraction Solution (Intron Biotechnology, Gyeonggi-Do, Korea), following the manufacturer’s instructions. Protein concentrations were determined using the Bradford reagent (Bio-Rad Laboratories, Hercules, CA, USA). Western blot analysis was performed to further confirm the responses using antibodies recognising specific epitopes. Proteins were separated by SDS-PAGE, transferred to nitrocellulose membranes, and detected with the following antibodies: anti-phosphorylated (phospho)-Thr^172^ AMPK (Cell Signaling Technology, Danvers, MA, USA), anti-total AMPK (Cell Signaling Technology), anti-peroxisome proliferator-activated receptor (PPAR) α (Abcam, Cambridge, UK), anti-PPARγ, anti-phospho-ACC (Santa Cruz Biotechnology, Santa Cruz, CA, USA), anti-total ACC (Santa Cruz Biotechnology), anti-sterol regulatory element-binding protein 1 (SREBP-1; Santa Cruz Biotechnology), anti-B cell leukaemia/lymphoma 2 (BCL-2; Santa Cruz Biotechnology), anti-BCL-2-associated X protein (BAX; Santa Cruz Biotechnology), and anti-β-actin (Sigma-Aldrich, St Louis, MO, USA). After washing, the membranes were incubated with anti-mouse IgG or anti-rabbit IgG horseradish peroxidase (HRP)-linked secondary antibodies (Cell Signaling Technology) or a rabbit anti-goat IgG HRP-peroxidase secondary antibody (Sigma-Aldrich). The membranes were developed using an ECL Plus Detection Kit (Amersham International, Buckinghamshire, UK) to produce chemiluminescence signals, which were captured on X-ray films. Band densities were quantified with Quantity One software (Bio-Rad Laboratories).

### Cell culture and small interfering RNA (siRNA) transfection

HGECs were purchased from Anigio-Proteomie (Boston, MA, USA) and subcultured in endo-growth media (Angio-Proteomie). After treatment with medium containing different concentrations of d-glucose (5 mmol/L d-glucose (low glucose), 40 mmol/L d-glucose (high glucose), or 5 mmol/L d-glucose plus 35 mmol/L mannitol (osmotic control)) and 1, 10, or 50 µg/mL anthocyanin for 6 h, western blotting was performed for phospho-Thr^172^ AMPK, total AMPK, PPARα, PGC-1α, ERR-1α, PPARγ, phospho-ACC, total ACC, superoxide dismutase (SOD)-1, SOD-2, BCL-2, BAX, and β-actin with specific antibodies. To examine the effects of anthocyanins on other renal cells in high-glucose medium, we also used cultured NMS2 mesangial cells (see Additional file 1 for further details).

siRNAs targeted toward Ampkα1, Ampkα2, and Sirt1, and a non-specific scrambled siRNA (siRNA control) were complexed with a transfection reagent (Lipofectamine 2000; Invitrogen, Carlsbad, CA, USA), according to the manufacturer’s instructions. The sequences of the siRNAs were: α1-AMPK, 5′-GCAUAUGCUGCAGGUAGAU-3′; α2-AMPK, 5′-CGUCAUUGAUGAUGAGGCU-3′; SIRT1, 5′-CACCUGAGUUGGAUGAUAU-3′; and scrambled siRNA, 5′-CCUACGCCACCAAUUUCGU-3′ (Bioneer, Daejeon, Korea). At 24 h after transfection, HGECs were exposed to 40 mmol/L glucose and 50 µg/mL anthocyanins for 24 h.

### Statistical analysis

Data are expressed as mean ± SD. Differences between groups were examined for statistical significance by ANOVA with the Bonferroni correction using SPSS version 19.0 (IBM Corp., Armonk, NY, USA). Values of *p* < 0.05 were considered statistically significant.

## Results

### Physical and biochemical characteristics of mice

Body weight was heavier in the diabetic mouse groups compared with the non-diabetic mouse groups (Table 1). Blood glucose, HbA_1C_, serum triglyceride, NEFA, total cholesterol, and amount of food eaten were higher in *db*/*db* mice than in *db*/*m* mice. *db*/*db* mice showed a significant increase in albuminuria compared with *db*/*m* mice. Anthocyanin treatment ameliorated serum NEFA, triglyceride, total cholesterol, and albuminuria in *db*/*db* mice (Table 1).

**Table 1:** Physical and biochemical characteristics of the control and diabetic mice with or without anthocyanin treatment at the end of the 12-week experimental period

	*db*/*m* control	*db*/*m* ATC	*db*/*db* control	*db*/*db* ATC
Body weight (g)	31.6 ± 2.2	31.3 ± 1.9	59.8 ± 5.7**	41.4 ± 5.1**
Kidney weight (g)	0.2 ± 0.01	0.2 ± 0.02	0.26 ± 0.01*	0.24 ± 0.04*
Food intake (g/d)	1.2 ± 1.1	1.3 ± 0.8	4.0 ± 0.1**	5.7 ± 2.1**
FBS (mg/dL)	136 ± 21	142 ± 17	577 ± 49**	552 ± 66**
HbA_1c_ (%)	4.1 ± 0.1	3.9 ± 0.3	13.0 ± 1.2**	11.8 ± 2.5**
NEFA (mEq/L)	1.01 ± 0.08	0.98 ± 0.08	1.92 ± 0.25**	1.04 ± 0.13
Triglycerides (mg/dL)	40.1 ± 8.9	20.8 ± 9.8	71.9 ± 13.7**	28.0 ± 20.0
Total cholesterol (mg/dL)	26.6 ± 5.9	30.3 ± 1.8	41.8 ± 9.4*	29.4 ± 2.6
24-h albuminuria (µg)	11.4 ± 2.9	4.4 ± 2.7	169.4 ± 74.5**	62.0 ± 37.0
Urine volume (mL)	0.4 ± 0.5	0.4 ± 0.4	10.6 ± 4.9**	10.7 ± 4.2**

Data are mean ± SD (n = 6–8 per group).

*FBS* fasting blood sugar.

* *p* < 0.05 and ** *p* < 0.001 compared with the other groups.

### Effects of anthocyanin on renal phenotype, TGF-β1, and type IV collagen

**Figure 1:**
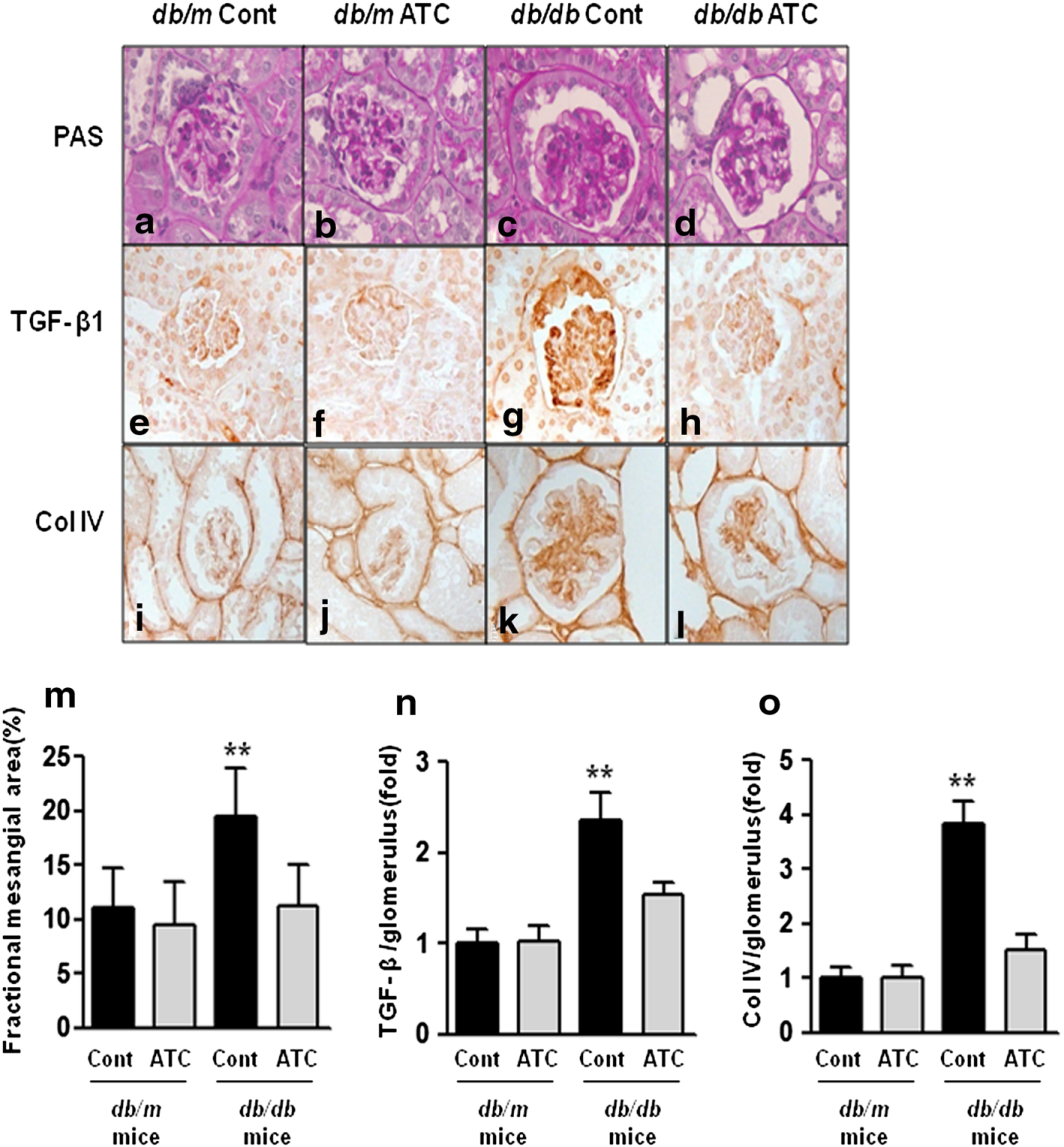
Changes in glomerular phenotypes in anthocyanin-treated *db*/*db* mice. Glomerular mesangial fractional area, TGF-β_1_ expression, and collagen IV expression in the cortical area of *db*/*m* and *db*/*db* mice, with or without anthocyanin treatment, were evaluated. **a**–**l** Representative sections stained with periodic acid-Schiff reagent and representative immunohistochemical staining for TGF-β1 and type IV collagen (original magnification ×400). **m**–**o** Quantitative analyses of the mesangial fractional area (%) (**m**), TGF-β1 (fold) (**n**), and type IV collagen (fold) (**o**). **p < 0.01 vs. *db*/*m* control, *db*/*m* ATC, and *db*/*db* ATC mice. *Col IV* type IV collagen, *PAS* periodic acid-Schiff stain.

There were no differences in the fractional mesangial areas between *db*/*m* and *db*/*m* anthocyanin mice (Figure 1a–d, m). There was a marked increase in the mesangial area in *db*/*db* mice compared with *db*/*m* mice (*p* < 0.01). Consistent with the changes in the mesangial fractional area, expression of the pro-fibrotic growth factor TGF-β1, which is associated with extracellular matrix type IV collagen, was significantly increased in *db*/*db* mice compared with *db/m* and *db/m* anthocyanin mice (Figure 1e–l, n–o). All diabetes-induced renal phenotypic changes and inflammation in *db*/*db* mice improved with anthocyanin treatment.

### Renal cortical expression of phospho-Thr^172^ AMPK, total AMPK, PPARα, PPARγ, total ACC, phospho-ACC, SREBP-1, and intra-renal NEFA, triglyceride, and total cholesterol

**Figure 2:**
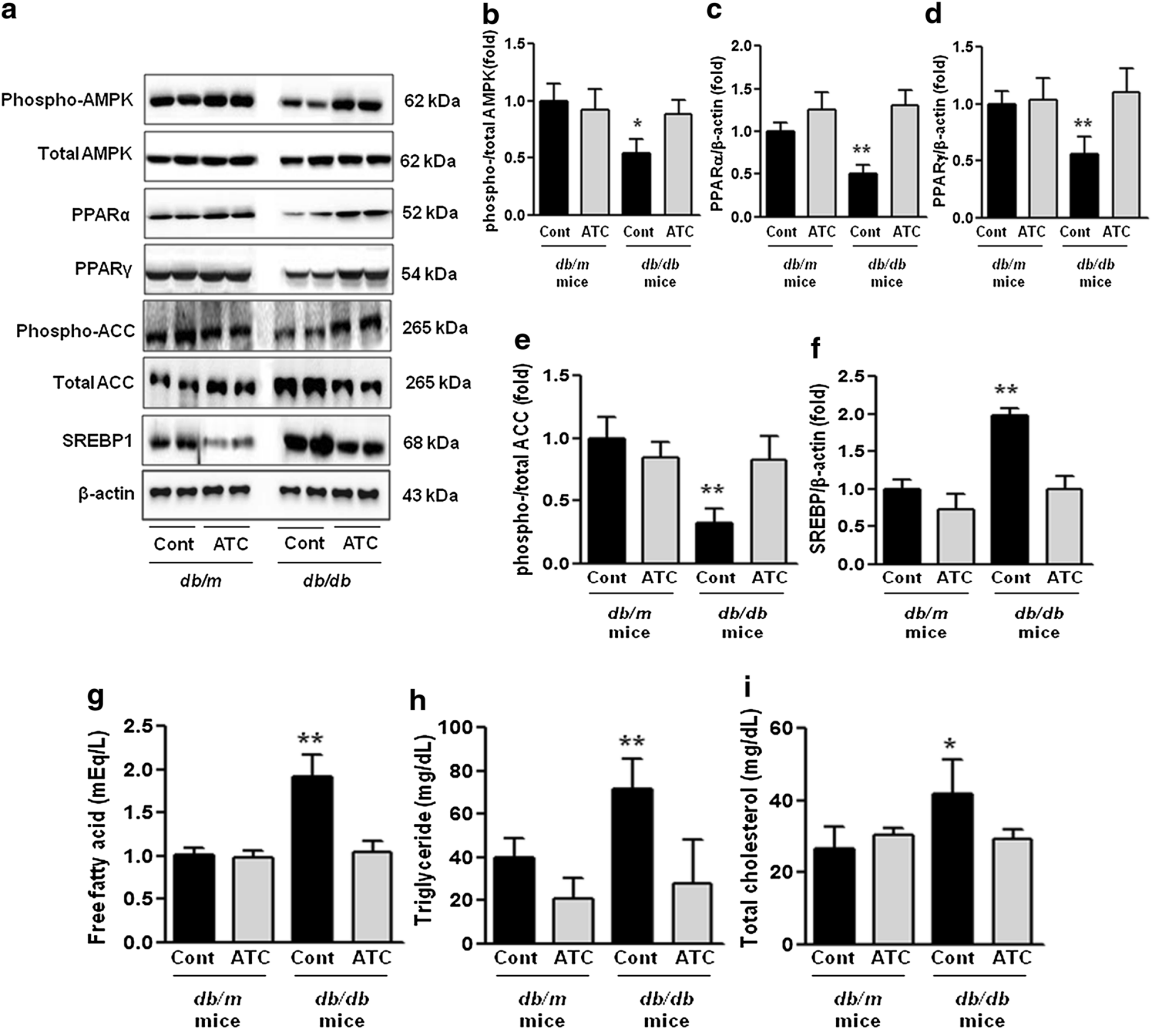
Phospho-Thr^172^ AMPK, total AMPK, PPARα, PPARγ, phospho-ACC, total ACC, SREBP-1, and intra-renal lipid levels in the renal cortex of *db*/*m* and *db*/*db* mice with or without anthocyanin treatment. Protein lysates (30 μg) from the renal cortex were separated by SDS-PAGE and analysed by western blotting. **a** Representative western blots for phospho-Thr^172^ AMPK, total AMPK, PPARα, PPARγ, phospho-ACC, total ACC, SREBP-1, and β-actin. **b**–**f** Quantitative analyses for phospho-Thr^172^ AMPK/total AMPK (**b**), PPARα/β-actin (**c**), PPARγ/β-actin (**d**), phospho-ACC/total ACC (**e**), and SREBP-1/β-actin (**f**). *p < 0.05 and **p < 0.01 *vs.* *db*/*m* control, *db*/*m* ATC, and *db/db* ATC mice. **g**–**i** Quantitative analyses of intra-renal NEFA (**g**), triglyceride (**h**), and total cholesterol (**i**) concentrations. *p < 0.05 and **p < 0.01 vs. *db*/*m* control, *db*/*m* ATC, and *db*/*db* ATC mice.

Western blotting showed that diabetes markedly decreased the phospho-AMPK Thr^172^/total AMPK ratio in *db*/*db* mice compared with *db*/*m* and *db*/*m* anthocyanin mice (Figure 2a). Anthocyanin treatment restored the phospho-AMPK Thr^172^/total AMPK ratio in *db*/*db* mice to the levels in *db*/*m* and *db*/*m* anthocyanin mice (Figure 2a, b; *p* < 0.05). The levels of PPARα and PPARγ were lower in *db*/*db* mice compared with *db*/*m* and *db*/*m* anthocyanin mice, as assessed by western blotting, and improved with anthocyanin treatment (Figure 2a, c, d; *p* < 0.01). The phospho-ACC/total ACC ratio and SREBP-1 level were evaluated to determine the changes in lipid metabolism as target proteins of AMPK. A decrease in the phospho-ACC/total ACC ratio and an increase in the SREBP-1 level were seen in *db/db* mice, and were restored with anthocyanin treatment (Figure 2a, e, f; *p* < 0.01).

Direct measurement of intra-renal lipid concentrations showed increases in the NEFA, triglyceride, and total cholesterol concentrations, consistent with PPARα, PPARγ, phospho-ACC/total ACC ratio, and SREBP-1, which were ameliorated with anthocyanin treatment (Figure 2g–i; *p* < 0.05).

### Renal expression of pro-apoptotic BAX, anti-apoptotic BCL-2, and TUNEL-positive cells

**Figure 3:**
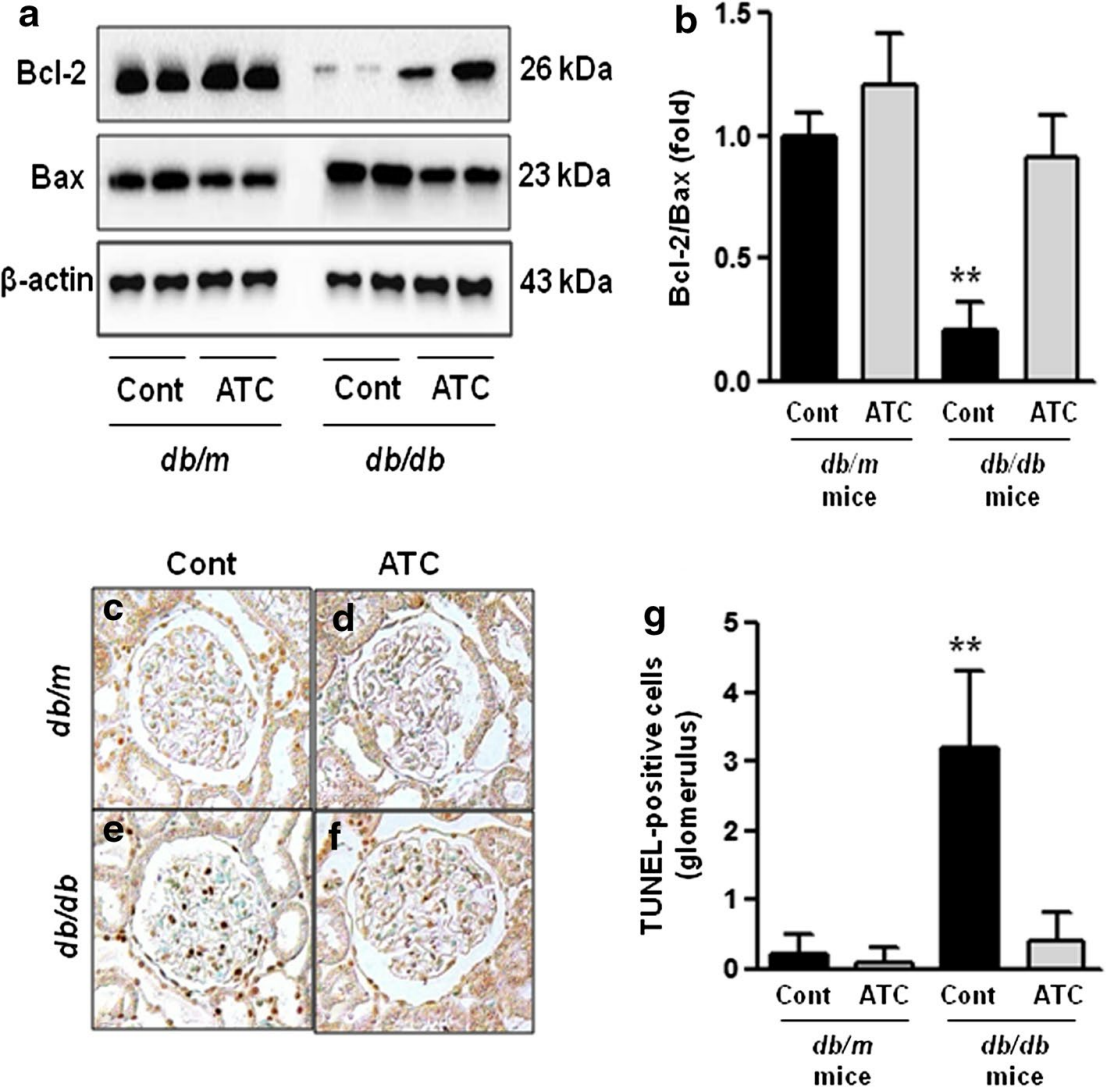
Anti-apoptotic BCL-2, pro-apoptotic BAX, and TUNEL assays in the renal cortex of *db/m* and *db/db* mice with or without anthocyanin treatment. Protein lysates (10 μg) from the renal cortex were separated by SDS-PAGE and analysed by western blotting. **a** Representative western blots of BCL-2, BAX, and β-actin levels. **b** Quantitative analyses of the BCL-2/BAX ratio. **c**–**g** Representative immunohistochemical staining for TUNEL-positive cells in *db*/*m* control (**c**), *db*/*m* ATC (**d**), *db*/*db* control (**e**), and *db*/*db* ATC (**f**) mice, and quantitative analyses of the results (**g**). **p < 0.01 vs. *db*/*m* control, *db*/*m* ATC, and *db*/*db* ATC mice.

The BAX protein levels were increased in *db*/*db* mice compared with *db*/*m* and *db*/*m* anthocyanin mice, as observed by western blotting (Figure 3a). The BCL-2 protein levels were decreased in *db/db* mice compared with *db*/*m* and *db*/*m* anthocyanin mice (Figure 3a). Consequently, the BCL-2/BAX ratio was significantly decreased in *db*/*db* mice. Anthocyanin treatment of *db*/*db* mice increased the BCL-2 protein levels and decreased the BAX protein levels, resulting in a normalised ratio of BCL-2/BAX expression (Figure 3b; *p* < 0.01).

There was a significant increase in the number of TUNEL-positive cells in the glomeruli of *db*/*db* mice compared with *db*/*m* and *db*/*m* anthocyanin mice. The number of TUNEL-positive cells in *db*/*db* mice was decreased after anthocyanin treatment (Figure 3c–g; *p* < 0.01).

### Effects of anthocyanin on renal 24-h urinary 8-isoprostane concentrations

**Figure 4:**
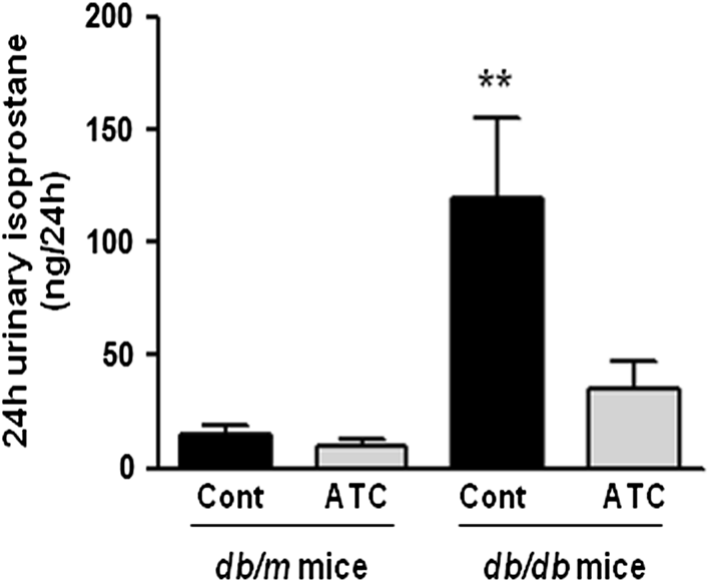
Twenty-four-hour urinary 8-isoprostane concentrations in *db*/*m* and *db*/*db* mice with or without anthocyanin treatment. The 24-h urinary 8-isoprostane concentrations of the mice are shown. **p < 0.01 vs. *db*/*m* control, *db*/*m* ATC, and *db*/*db* ATC mice.

Increases in renal oxidative stress and lipid peroxidation were observed in *db*/*db* mice compared with *db*/*m* and *db*/*m* anthocyanin mice with respect to urinary 8-isoprostane concentrations (Figure 4; *p* < 0.01). The 24-h urinary 8-isoprostane concentration was significantly decreased in *db*/*db* mice with anthocyanin treatment compared with the control groups. Taken together, these findings suggest that oxidative stress in *db*/*db* mice could be ameliorated by anthocyanin treatment.

### In vitro studies

**Figure 5:**
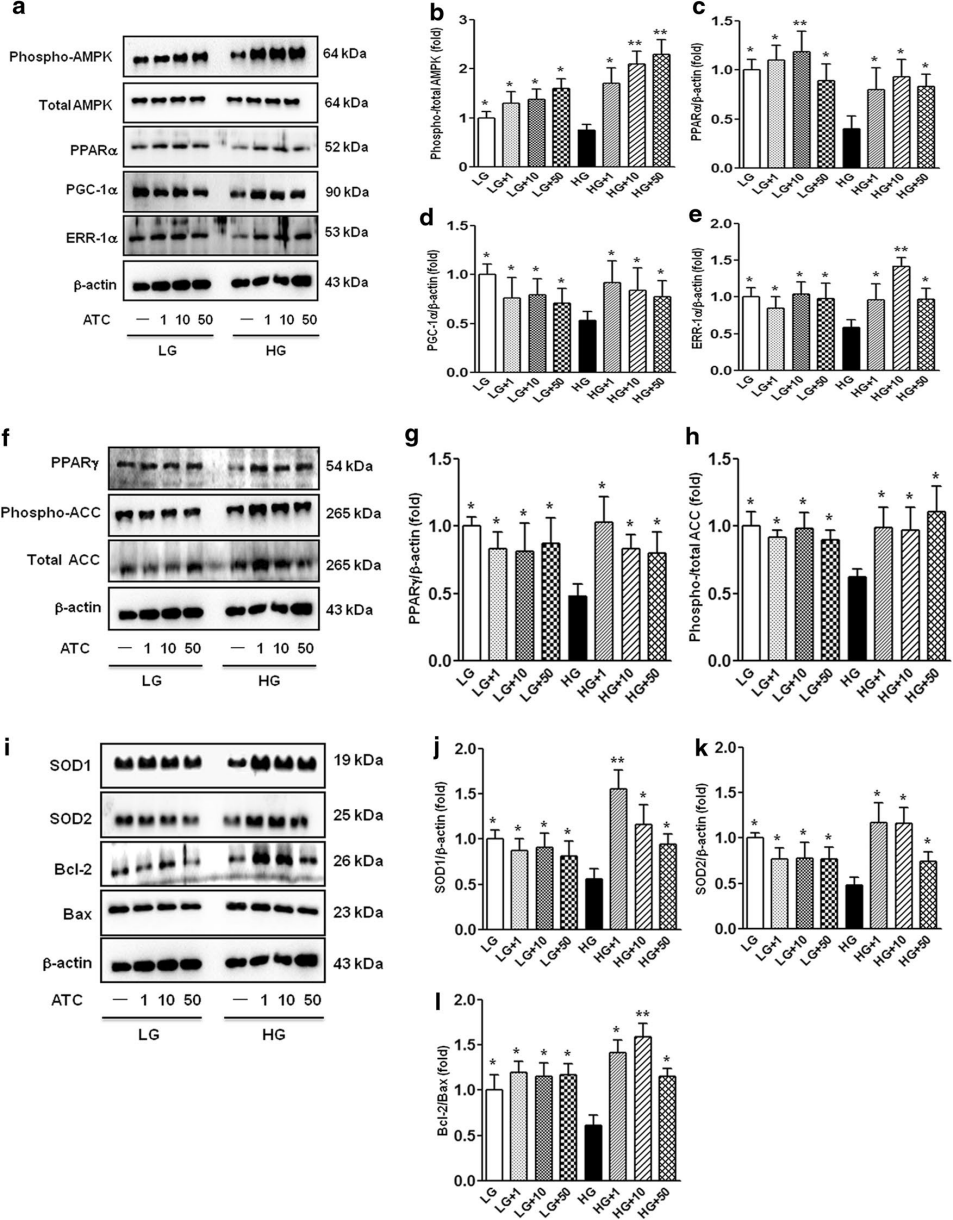
Effect of anthocyanins on intracellular signalling, apoptosis, and oxidative stress in HGECs cultured in low-glucose (5 mmol/L d-glucose) or high-glucose (40 mmol/L d-glucose) medium with or without anthocyanin treatment (50 μg/mL). Phospho-Thr^172^ AMPK, total AMPK, PPARα, PGC-1α, ERR-1α, PPARγ, phospho-ACC, total ACC, and β-actin levels were assessed using cultured HGECs. Protein lysates (10 μg) were separated by SDS-PAGE and analysed by western blotting. **a** Representative western blots and quantitative analyses of phospho-Thr^172^ AMPK, total AMPK, PPARα, PGC-1α, ERR-1α, and β-actin. **b**–**e** Quantitative analyses of phospho-Thr^172^ AMPK/total AMPK (**b**), PPARα (**c**), PGC-1α (**d**), and ERR-1α (**e**). **f**–**h** Representative western blots of PPARγ, phospho-ACC, total ACC, and β-actin in HGECs (**f**) and quantitative analyses of the results **g**, **h**. **i**–**l** Representative western blots of SOD-1, SOD-2, BCL-2, BAX, and β-actin levels in HGECs (**i**) and quantitative analyses of the results (**j**–**l**). *p < 0.05 and **p < 0.01 compared with high-glucose medium. *HG* high glucose, *LG* low glucose.

We evaluated the effects of anthocyanins on high glucose-induced oxidative stress and apoptosis related to AMPK activation and its downstream molecules in cultured HGECs. High glucose (40 mmol/L d-glucose) induced significant decreases in the activation of phosphor-Thr^172^ AMPK (Figure 5a, b) and PPARα, PGC-1α, and ERR-1α levels (Figure 5a, c–e). Consistent with these results, high glucose decreased the PPARγ levels and inactivated ACC (Figure 5f–h). High glucose also decreased SOD-1, SOD-2, and anti-apoptotic activity of BCL-2/BAX (Figure 5i–l). In contrast, anthocyanins prevented high glucose-induced oxidative stress and apoptosis related to the activation of AMPK, PPARα–PGC-1α–ERR-1α signalling, PPARγ, and inactivation of ACC. When compared with high-glucose medium, addition of anthocyanin to low-glucose medium did not affect intracellular signalling and glomerular endothelial cells.

**Figure 6:**
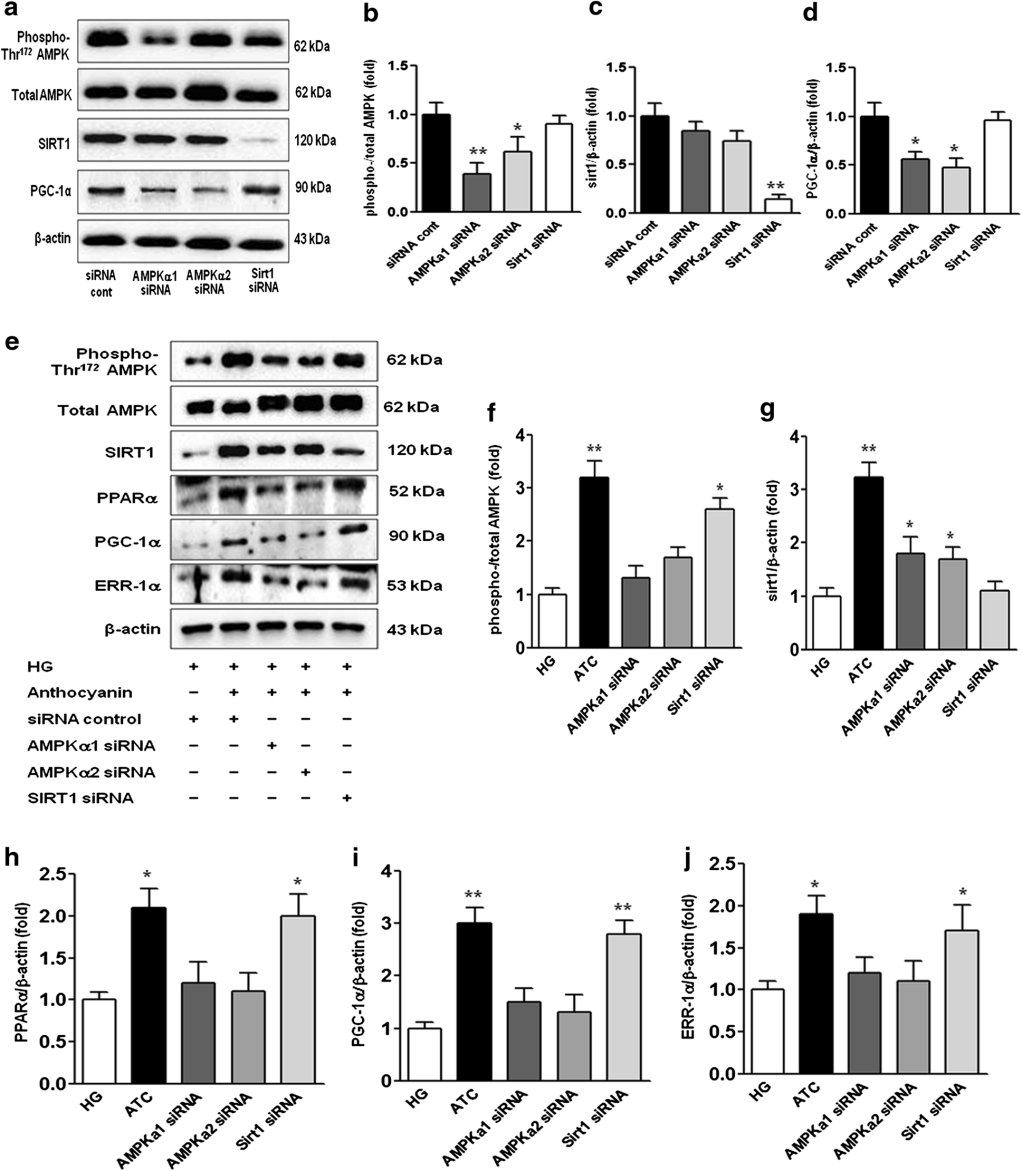
Effect of *AMPKα1*, *AMPKα2*, and *SIRT1* siRNAs on anthocyanin-stimulated AMPK signalling in HGECs. Protein lysates (10 μg) from cultured HGECs were separated by SDS-PAGE and analysed by western blotting. Cultured HGECs were transfected with 40 nmol/L control siRNA or 40 nmol/L *AMPKα1*, *AMPKα2*, or *SIRT1* siRNAs using a transfection reagent (Lipofectamine 2000). At approximately 24 h after transfection, the levels of phospho-AMPK Thr^172^, total AMPK, SIRT1, and PGC-1α signalling in low-glucose medium were analysed. **a**–**d** Representative western blots (**a**) and quantitative analyses of the results **b**–**d**. *p < 0.05 and **p < 0.01 compared with the other groups. Cultured HGECs were transfected with 50 nmol/L control siRNA or 50 nmol/L *AMPKΑ1*, *AMPKΑ2*, or *SIRT1* siRNAs and stimulated with anthocyanin in high-glucose medium. At approximately 24 h after stimulation, the levels of phospho-AMPK Thr^172^, total AMPK, SIRT1, PPARα, PGC-1α, and ERR-1α signalling in high-glucose medium were analysed. **e**–**j** Representative western blots of phospho-AMPK Thr^172^, total AMPK, PPARα, PGC-1α, ERR-1α, and β-actin levels (**e**) and quantitative analyses of the results (**f**–**j**). *p < 0.05 and **p < 0.01 compared with high-glucose medium. *HG* high glucose.

To show whether the anti-apoptotic effect of anthocyanins is AMPK-dependent in diabetes, we performed additional experiments using siRNAs for *AMPKΑ1*, *AMPKΑ2*, and *SIRT1* in cultured HGECs (Figure 6a–d). Transfected siRNAs for *AMPKΑ1* and *AMPKΑ2* suppressed anthocyanin-induced AMPK and PPARα–PGC-1α–ERR-1α signalling compared with the siRNA control group. However, transfection with the siRNA for *Sirt1* only suppressed SIRT1, and not the phospho-Thr^172^ AMPK/total AMPK ratio and PPARα–PGC-1α–ERR-1α signalling (Figure 6e–j).

## Discussion

The present results suggest that anthocyanin-rich SE ameliorated renal function and phenotype in the examined mouse model of type 2 diabetes, and was related to renal lipid accumulation, apoptotic renal cell injury, and oxidative stress through restoration of decreased AMPK activity and its target molecules in diabetic nephropathy. Downstream regulators of AMPK including ACC, SREBP-1, and PPAR were all translated into restoration of diabetes-induced lipotoxicity by anthocyanins in the mouse model and in vitro experiments. Using siRNAs for *AMPKΑ1* and *AMPKΑ2* in HGECs, we observed beneficial effects of anthocyanins on lipotoxicity in an AMPK-dependent manner.

The ultrasensitive energy sensor AMPK activates the catabolic pathway, inhibits the anabolic pathway, and restores energy homeostasis by phosphorylating multiple substrates in many metabolic syndrome-associated diseases [13, 15, 22, 23]. Among the target molecules of AMPK, ACC is related to fatty acid oxidation and inhibition of fatty acid synthesis in many organs, such as the liver and kidneys. Phosphorylation of ACC1 at Ser^79^ and ACC2 at Ser^218^ by AMPK leads to inhibition of ACC activity and decreased malonyl-CoA, which is pivotal in controlling the rate of fatty acid β-oxidation. It has been reported that the reduced AMPK activity in diabetic nephropathy is linked to increased triglyceride accumulation because of reduced inhibitory phosphorylation of ACC [15, 24].

SREBP, another target molecule controlled by AMPK, is a transcription factor regulating cellular lipogenesis and lipid homeostasis. Mammalian SREBP has three isoforms (SREBP-1a, SREBP-1c, and SREBP-2) and distinct but overlapping lipogenic transcriptional processes [25]. The nutrient sensor AMPK negatively regulates SREBP to limit lipogenesis by directly phosphorylating SREBP. In our previous studies, we found that suppressed AMPK and the subsequent increase in SREBP activity in diabetic nephropathy lead to lipotoxicity in the pathogenesis of diabetic nephropathy [26, 27]. In the present study, anthocyanin-mediated AMPK activation increased phosphorylation of ACC and decreased SREBP, which were correlated with decreased lipid contents in the diabetic kidney.

PPARs have noteworthy biological functions as sensors for fatty acid derivatives and control important metabolic pathways involved in lipid and energy metabolism [28, 29]. Renal PPARα and PPARγ play important roles in modulating energy utilisation in the kidney through regulation of renal fatty acid oxidation. Stimulation of fatty acid oxidation through PPAR activation provides a potential mechanism by which the lipid content of tissues can be reduced to prevent lipid accumulation. In light of this, PPAR upregulation by anthocyanins seems to be another therapeutic target for improvement of lipotoxicity in diabetic nephropathy [30–32].

Our in vitro study demonstrated that another critical mediator of the effects of PPARα on lipotoxicity is PGC-1α, a coactivator protein that interacts with several transcription factors, including ERR-1α, an important mediator in mitochondrial biogenesis. Notably, ERR-1α activated by PGC-1α induces genes that play roles in lipid transport and fatty acid oxidation [33]. Meanwhile, SIRT1 is another molecule that responds to a variable nutrient status and is considered an AMPK partner [34]. The results for the siRNA for *SIRT1* in the present in vitro study suggest that the effect of anthocyanins operates in a SIRT1-independent pathway.

The mechanism of lipotoxic injury in diabetic nephropathy could be explained by downregulation of fatty acid oxidation with altered expression related to energy sensor AMPK activity and its downstream lipogenic transcription factors, including PPAR and SREBP-1 [2]. Although this may not entirely explain the complex pathogenesis of diabetic nephropathy, it has not been explored to its fullest potential. Recently, some investigators reported that altered renal lipid metabolism and accumulation in diabetic nephropathy were observed in humans [1]. They clarified that heavy lipid deposition and increased intracellular lipid droplets were associated with dysregulation of lipid metabolism genes, and suggested that renal lipid metabolism may serve as a target for specific therapies of human diabetic nephropathy [1, 26].

Anthocyanins are natural phytochemicals and the most oxidized flavonoids with a fully unsaturated C ring and a hydroxyl at position 3. They can be found as glycosylated forms of polyhydroxy and polymethoxy derivatives of 2-phenylbenzopyrylium, acylated or non-acylated with aliphatic acids, thus providing a rich source of potential therapeutic agents as antioxidants. Most experimental data have verified that anthocyanins serve as antioxidants and have anti-inflammatory properties in metabolic diseases. Takikawa et al. showed that anthocyanin-rich bilberry extract ameliorates hyperglycaemia and insulin sensitivity via AMPK activation in white adipose tissue, skeletal muscle, and the liver in type 2 diabetic mice [17, 35]. These effects were accompanied by inactivation of ACC and upregulation of decreased PPARα expression. Valenti et al. [4] proposed a possible mechanism for the beneficial effects of anthocyanins in non-alcoholic fatty liver disease and non-alcoholic steatohepatitis. They explained that the effect of anthocyanins on lipid metabolism is dependent on activation of the AMPK pathway and its target molecules, SREBP-1c and PPARα, in hepatocytes. In the present study, we found that diabetic nephropathy exhibited profound metabolic abnormalities, including increased intra-renal lipid accumulation in kidney tissue as well as circulating NEFA and triglyceride concentrations, while all levels were restored with anthocyanin treatment via AMPK activation and its target molecules such as ACC, SREBP-1, and PPAR. Taken together, these results demonstrated that anthocyanins improved decreased AMPK activity and the accompanying lipotoxicity in the examined mouse model of diabetes. Further studies are needed to verify the mechanisms.

It is uncertain whether the target of anthocyanins is solely AMPK or whether it overlaps with upstream molecules, such as adiponectin. Little is known about the roles of upstream molecules of AMPK such as adiponectin, LKB1, and CaMKKβ in the regulation of AMPK in the kidney [36]. Recently, Liu et al. [3] reported that anthocyanins enhance adiponectin secretion from adipose tissue and ameliorate diabetes-related endothelial dysfunction. Accordingly, we cannot exclude the possibility that anthocyanins restore decreased AMPK activity, in part, through increased activity of adiponectin [3, 16]. Furthermore, we could not determine the specific contributions of the bioactive compounds in SE to the observed effects or the amounts of these compounds incorporated into the kidney. As cyanidin-3-O-glucoside was the most abundant anthocyanin in SE, we cautiously supposed that the main therapeutic effect of SE was mediated by cyanidin-3-O-glucoside based on previous studies [9, 31], but future investigations are required to verify this notion.

We used glomerular endothelial cells under high-glucose conditions in an in vitro study. The results showed that the earliest indicator of kidney damage in diabetes is albuminuria, which is widely considered to reflect underlying endothelial dysfunction [18]. The overall effect of AMPK in the endothelium has been proposed as the potential improvement of endothelial dysfunction, although the specific roles of endothelial AMPK in the kidney have not been shown [36]. Considering the marked differences in the metabolic profiles of the individual cell types within the kidney, it could be important to identify the differences in AMPK expression and the effects of anthocyanins on other renal cell populations, such as podocytes. The results for cultured mesangial cells in high-glucose medium with anthocyanin treatments showed similar patterns in the present study (Additional file 2).

In conclusion, reduced AMPK activity was associated with lipotoxicity, which could be related to apoptosis and oxidative stress in diabetic nephropathy. Anthocyanins, as new and interesting AMPK activators, attenuated lipotoxicity through inhibition of ACC and SREBP-1 and restoration of PPARα and PPARγ, which mediated the removal of lipid accumulation in the kidney. This was accompanied by improvement of glomerular matrix accumulation and albuminuria. The data suggest that high glucose-induced HGEC damage translated into reduced AMPK activity and exacerbated oxidative stress and apoptotic cell damage in the diabetic setting. All changes were reversed with anthocyanin treatment.

## Conclusions

The results of the present study demonstrated that anthocyanin-rich SE ameliorated diabetic nephropathy in *db*/*db* mice via AMPK activation and the consequent effects on its target molecules, which appeared to prevent lipotoxicity-related apoptosis and oxidative stress in the kidney. In this manner, anthocyanins may have a potential therapeutic role in type 2 diabetic nephropathy. Further clinical investigations are needed to evaluate the precise molecular mechanisms of the bioactive compounds, including anthocyanins, in SE.

## Additional files

**Additional file 1:** Method using cultured mesangial cells in vitro study.
**Additional file 2:** Result of anthocyanin effect on intracellular signalling in the cultured mesangial cells.

## Abbreviations

ACC: acetyl-CoA carboxylase
AMPK: adenosine monophosphate-activated protein kinase
ATC: anthocyanin
BAX: BCL-2-associated X protein
BCL-2: B cell leukaemia/lymphoma-2
CaMKKβ: calcium/calmodulin-dependent protein kinase kinase β
Col IV: type IV collagen
ERR-1α: oestrogen-related receptor-1α
HGEC: human glomerular endothelial cell
LKB1: liver kinase B1
PGC-1α: PPARγ coactivator-1α
PPAR: peroxisome proliferator-activated receptor
SE: Seoritae extract
si: small interfering
SIRT1: silent information regulator T1
SOD: superoxide dismutase
SREBP-1: sterol regulatory element-binding protein-1
TGF-β1: transforming growth factor-β1.
