# Supplementary material for: Anthocyanin-rich Seoritae extract ameliorates renal lipotoxicity via activation of AMP-activated protein kinase in diabetic mice
Source: J Transl Med. 2015 Jun 27;13:203. doi: 10.1186/s12967-015-0563-4 (PMC4482313; doi:10.1186/s12967-015-0563-4)
Supplement: Supplementary file 1 — Additional file 1: Method using cultured mesangial cells in vitro study. [file 12967_2015_563_MOESM1_ESM.docx]

**ESM methods**

*In vitro study in cultured mesangial cells*

Rat normal mesangial cells (NMS) cells were maintained in RPMI 1640 medium with 11.1 mmol/L of glucose, supplemented with heat-inactivated 10% FBS (Hyclone, Logan, UT, USA). To find the effect of anthocyanin on mesangial cell in high-glucose media, mesangial cells were grown in 5 mmol/L D-glucose, 40 mmol/L D-glucose or 5 mmol/L D-glucose plus 35 mmol/L mannitol (as an osmotic control for 40 mmol/L D-glucose) without or with an additional 6-h application of anthocyanin (1, 10 or 50 ng/mL, respectively). Western blot analysis was performed for phosphorylated (phospho)-Thr^172^ AMPK (Cell Signaling Technology, Danvers, MA, USA), total AMPK (Cell Signaling Technology), PPARα (Abcam, Cambridge, UK), PPARγ (Abcam), phosphorylated ACC (Santa Cruz Biotechnology, Santa Cruz, CA, USA) and β-actin (Sigma-Aldrich, St Louis, MO, USA) with specific antibodies.
