# Supplementary material for: Anthocyanin-rich Seoritae extract ameliorates renal lipotoxicity via activation of AMP-activated protein kinase in diabetic mice
Source: J Transl Med. 2015 Jun 27;13:203. doi: 10.1186/s12967-015-0563-4 (PMC4482313; doi:10.1186/s12967-015-0563-4)
Supplement: Supplementary file 2 — Additional file 2: Result of anthocyanin effect on intracellular signalling in the cultured mesangial cells. [file 12967_2015_563_MOESM2_ESM.pptx]

## Slide 1
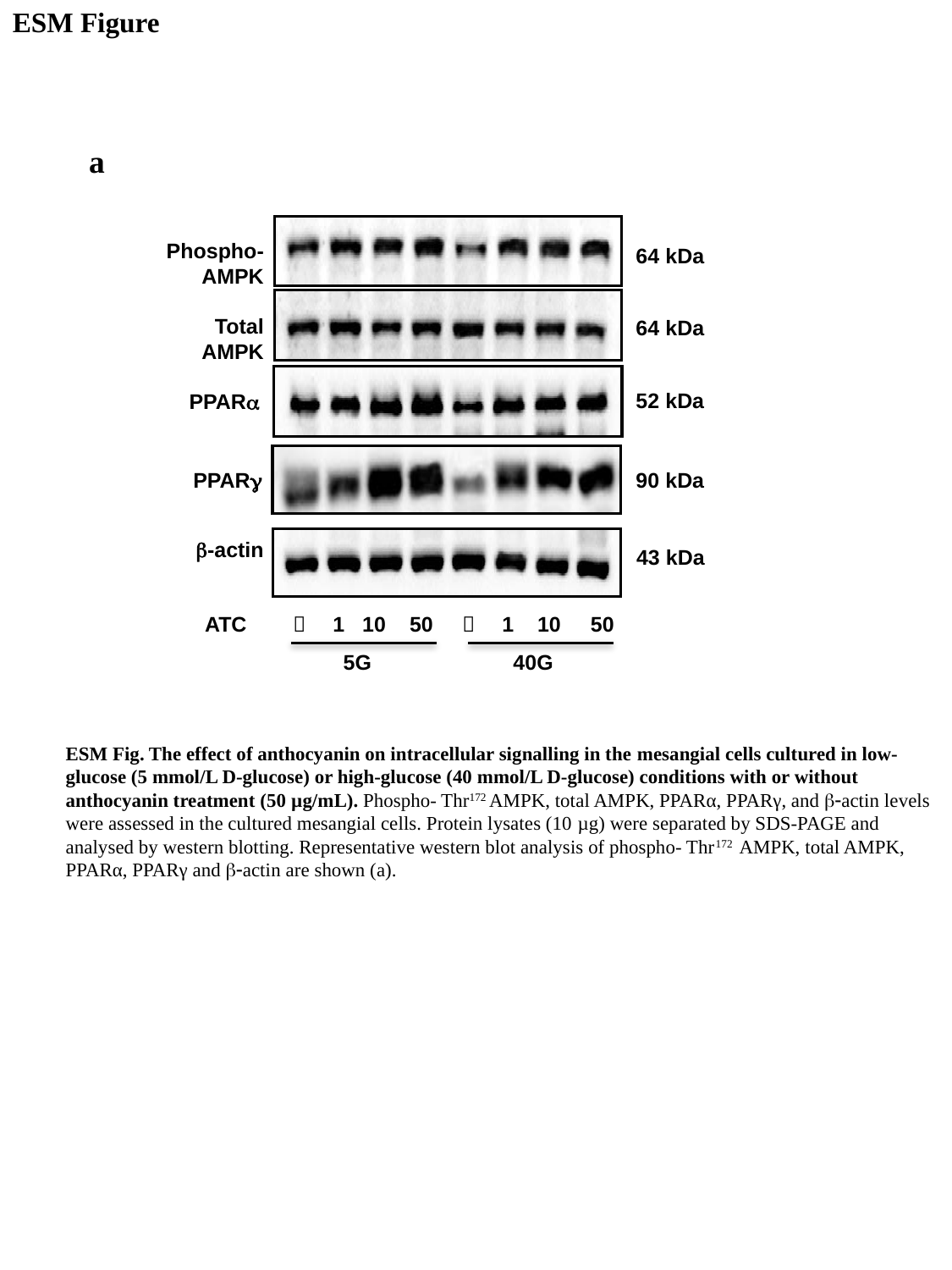

ESM Figure
a
Phospho-AMPK
64 kDa
Total AMPK
64 kDa
52 kDa
PPARa
PPARγ
90 kDa
b-actin
43 kDa
 ATC ㅡ 1 10 50 ㅡ 1 10 50
 5G 40G
ESM Fig. The effect of anthocyanin on intracellular signalling in the mesangial cells cultured in low-glucose (5 mmol/L D-glucose) or high-glucose (40 mmol/L D-glucose) conditions with or without anthocyanin treatment (50 µg/mL). Phospho- Thr172 AMPK, total AMPK, PPARα, PPARγ, and b-actin levels were assessed in the cultured mesangial cells. Protein lysates (10 µg) were separated by SDS-PAGE and analysed by western blotting. Representative western blot analysis of phospho- Thr172 AMPK, total AMPK, PPARα, PPARγ and b-actin are shown (a).

## Slide 2
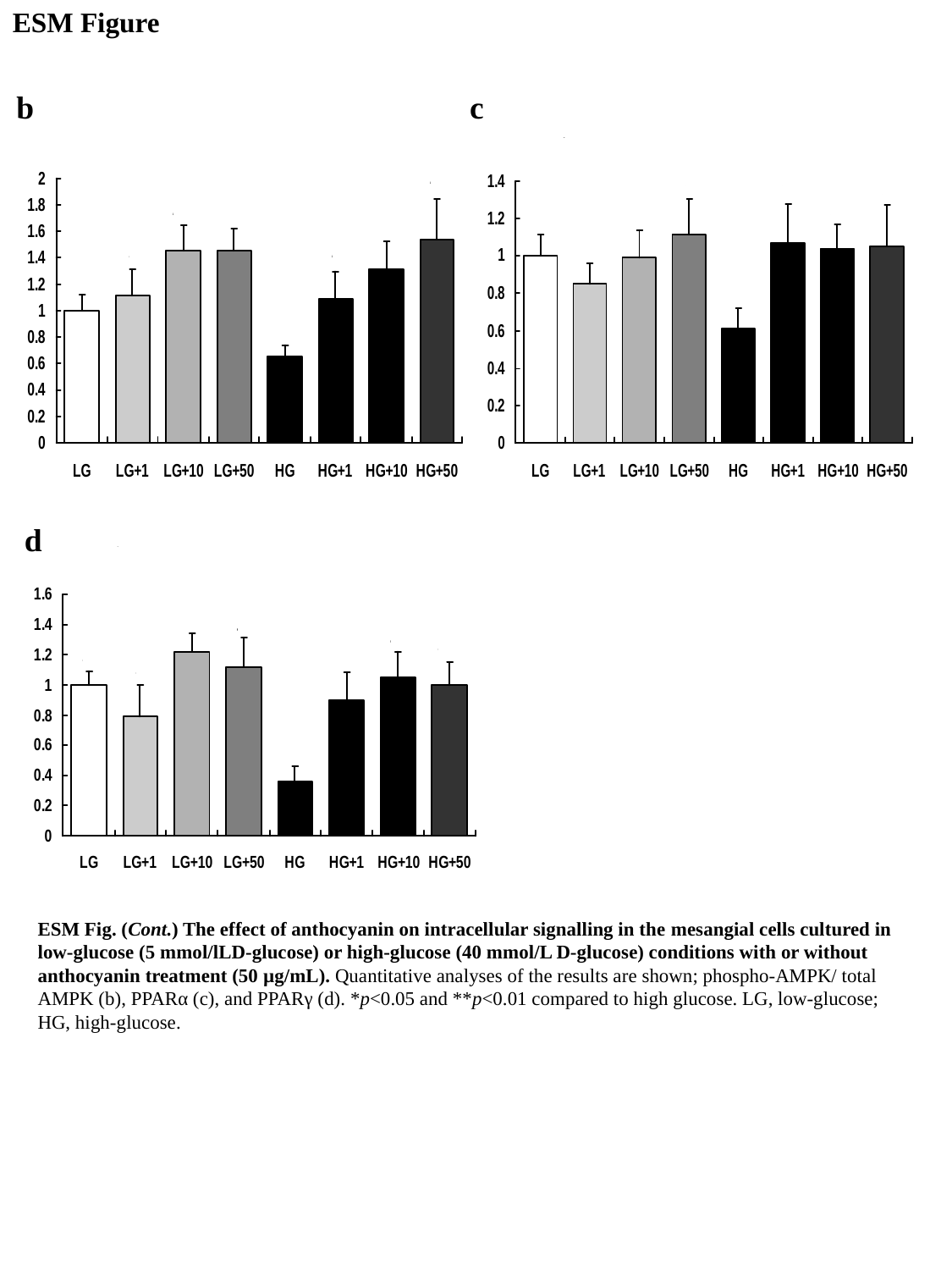

ESM Figure
b
c
d
ESM Fig. (Cont.) The effect of anthocyanin on intracellular signalling in the mesangial cells cultured in low-glucose (5 mmol/lLD-glucose) or high-glucose (40 mmol/L D-glucose) conditions with or without anthocyanin treatment (50 µg/mL). Quantitative analyses of the results are shown; phospho-AMPK/ total AMPK (b), PPARα (c), and PPARγ (d). *p<0.05 and **p<0.01 compared to high glucose. LG, low-glucose; HG, high-glucose.
